# Practical Notes on Diagnosis and Treatment

**Published:** 1906-10-13

**Authors:** 


### PRACTICAL NOTES ON DIAGNOSIS AND TREATMENT.

**Nocturnal Epilepsy.**

When the fits occur during the night, or soon after rising, measures which tend to keep up arterial tone should be adopted. A glass of hot milk or a cup of beef-tea, at bed-time in the one case and on waking in the other, would be in the right direction. Even a more definite stimulant may be added, as aromatic spirit of ammonia, to the milk, or a little cayenne pepper to the beef-tea.—*Sir William Broadbent.*

**Bladder Symptoms in Disseminated Sclerosis.**

A defect in the functions of the bladder is a frequent event in disseminated sclerosis. Sometimes it takes the form of incontinence, sometimes of difficulty in starting in the act of micturition. Indeed, the bladder disability may be, and not infrequently is, the first evidence of the disease, and, like many other symptoms, it may entirely and completely disappear.—*Dr. James Taylor.*

**Courvoisier's Law.**

"In chronic jaundice due to blocking of the common duct a contraction of the gall-bladder signifies that the obstruction is due to stone, a dilatation of the gall-bladder that it is due to causes other than stone." This rule is often of considerable clinical importance, as, for example, in the diagnosis between gall-stone and malignant disease. Exceptions doubtless occur, but they are few in number.

**Mercury in Myelitis.**

In myelitis, whether the patient be suffering from syphilis or not, there is no treatment that can compare with mercury, and mercury rubbed in is to be preferred to mercury given by the mouth. Whether rubbed in to other parts or not, it ought to be rubbed along the spinal column.—*Dr. Risien Russell.*

**Pyorrhœa Alveolaris.**

The essential principles of treatment are the removal of tartar and persevering cleanliness. A suitable mouth-wash is zinc sulphate 1 drachm, glycerine of carbolic acid 1 ounce, water 10 ounces. Use ½ ounce in a wineglassful of water night and morning.

**Age Incidence of Migraine.**

People do not begin to suffer from migraine in middle life. Indeed, this disorder tends as this period of life approaches to decline. Hence the appearance of symptoms suggesting migraine in a middle-aged patient should be viewed with suspicion.

**Enlarged Cervical Glands.**

A glandular enlargement at the root of the neck on the left side may be an expression of malignant disease of the œsophagus or even of the stomach. This fact may be of considerable value in the diagnosis of a doubtful case.

